# Supplementary material for: Modeling glioblastoma heterogeneity as a dynamic network of cell states
Source: Mol Syst Biol. 2021 Sep 16;17(9):e10105. doi: 10.15252/msb.202010105 (PMC8444284; doi:10.15252/msb.202010105)
Supplement: Supplementary file 5 — Source Data for Figure 3 [file MSB-17-e10105-s001.zip › Figure3A_sourcedata/GSEA_3065/hallmarks_state3.GseaPreranked.1623416274744/HALLMARK_ANGIOGENESIS.html]

Details for gene set HALLMARK\_ANGIOGENESIS[GSEA]

|  || Dataset | state3 |
| Phenotype | NoPhenotypeAvailable |
| Upregulated in class | na\_pos |
| GeneSet | HALLMARK\_ANGIOGENESIS |
| Enrichment Score (ES) | 0.75674015 |
| Normalized Enrichment Score (NES) | 2.1465406 |
| Nominal p-value | 0.0 |
| FDR q-value | 0.0 |
| FWER p-Value | 0.0 |
Table: GSEA Results Summary

  

Fig 1: Enrichment plot: HALLMARK\_ANGIOGENESIS      
 Profile of the Running ES Score & Positions of GeneSet Members on the Rank Ordered List

  

| PROBE | GENE SYMBOL | GENE\_TITLE | RANK IN GENE LIST | RANK METRIC SCORE | RUNNING ES | CORE ENRICHMENT || 1 | POSTN |  |  | 24 | 0.487 | 0.1152 | Yes |
| 2 | TIMP1 |  |  | 35 | 0.470 | 0.2277 | Yes |
| 3 | APP |  |  | 49 | 0.437 | 0.3319 | Yes |
| 4 | LPL |  |  | 68 | 0.381 | 0.4219 | Yes |
| 5 | LRPAP1 |  |  | 81 | 0.361 | 0.5079 | Yes |
| 6 | COL5A2 |  |  | 266 | 0.255 | 0.5484 | Yes |
| 7 | VCAN |  |  | 293 | 0.245 | 0.6047 | Yes |
| 8 | ITGAV |  |  | 322 | 0.238 | 0.6591 | Yes |
| 9 | FGFR1 |  |  | 419 | 0.215 | 0.7000 | Yes |
| 10 | FSTL1 |  |  | 790 | 0.159 | 0.6960 | Yes |
| 11 | PTK2 |  |  | 841 | 0.153 | 0.7274 | Yes |
| 12 | JAG1 |  |  | 1011 | 0.138 | 0.7414 | Yes |
| 13 | CCND2 |  |  | 1286 | 0.116 | 0.7380 | Yes |
| 14 | SPP1 |  |  | 1358 | 0.111 | 0.7567 | Yes |
| 15 | VEGFA |  |  | 1723 | 0.090 | 0.7369 | No |
| 16 | VAV2 |  |  | 3078 | 0.039 | 0.5913 | No |
| 17 | PDGFA |  |  | 3998 | 0.015 | 0.4897 | No |
| 18 | MSX1 |  |  | 5833 | -0.034 | 0.2879 | No |
| 19 | STC1 |  |  | 5905 | -0.036 | 0.2884 | No |
| 20 | TNFRSF21 |  |  | 7822 | -0.155 | 0.1064 | No |
Table: GSEA details [plain text format]

  

Fig 2: HALLMARK\_ANGIOGENESIS: Random ES distribution      
 Gene set null distribution of ES for **HALLMARK\_ANGIOGENESIS**
